# Antioxidant Capacity Determination of Hungarian-, Slovak-, and Polish-Origin Goldenrod Honeys †

**DOI:** 10.3390/plants11060792

**Published:** 2022-03-16

**Authors:** Szilvia Czigle, Rita Filep, Ema Balažová, Hajnalka Szentgyörgyi, Viktória Lilla Balázs, Marianna Kocsis, Dragica Purger, Nóra Papp, Ágnes Farkas

**Affiliations:** 1 Department of Pharmacognosy and Botany, Faculty of Pharmacy, Comenius University Bratislava, Odbojárov 10, 832-32 Bratislava, Slovakia; 2 Department of Pharmacognosy, Faculty of Pharmacy, University of Pécs, Rókus u. 2., 7624 Pecs, Hungary; filep.rita@pte.hu (R.F.); viktoria.balazs@aok.pte.hu (V.L.B.); dragica@gamma.ttk.pte.hu (D.P.); nora4595@gamma.ttk.pte.hu (N.P.); 3 Department of Cell and Molecular Biology of Drugs, Faculty of Pharmacy, Comenius University Bratislava, Odbojárov 10, 832-32 Bratislava, Slovakia; balazova168@uniba.sk; 4 Department of Plant Ecology, Institute of Botany, Faculty of Biology, Jagiellonian University, ul. Gronostajowa 3, 30-387 Krakow, Poland; hajnalka.szentgyorgyi@uj.edu.pl; 5 Department of Plant Biology, Institute of Biology, Faculty of Science, University of Pécs, Ifjúság u. 6., 7624 Pecs, Hungary; mkocsis@gamma.ttk.pte.hu

**Keywords:** unifloral honey, *Solidago*, DPPH, ABTS, FRAP, Pfund scale, pollen analysis, total polyphenols, flavonoids, phenolic acids

## Abstract

The goldenrod (*Solidago*) species are flowering plants that produce nectar and can be the sources of unifloral honeys. *S. canadensis* and *S. gigantea* are native to North America and invasive in several European countries, while *S. virgaurea* is native to Europe. The aim of this work was to determine and compare the antioxidant capacity of goldenrod honeys collected in three central European countries (Hungary, Poland, and Slovakia), from three locations within each country. The botanical origin of each honey sample was checked with melissopalynological analysis. Color intensity was determined using the Pfund scale. The antioxidant activity was determined with different spectrophotometric methods (DPPH, ABTS, and FRAP). The content of total polyphenols, flavonoids, and phenolic acids was quantified using spectrophotometric methods. The highest radical-scavenging activity was identified for Hungarian samples with all three antioxidant capacity assays. Medium antioxidant activity was described for Slovak samples. The DPPH and ABTS assays discriminated Polish honeys with the lowest antioxidant activity. The highest flavonoid and phenolic acid content was detected in Hungarian and Slovak honeys, while the lowest values were measured in Polish samples. Our study shows that the antioxidant capacity of unifloral goldenrod honeys can be different in various countries of origin, correlating with color intensity and polyphenol content.

## 1. Introduction

**Figure 1:**
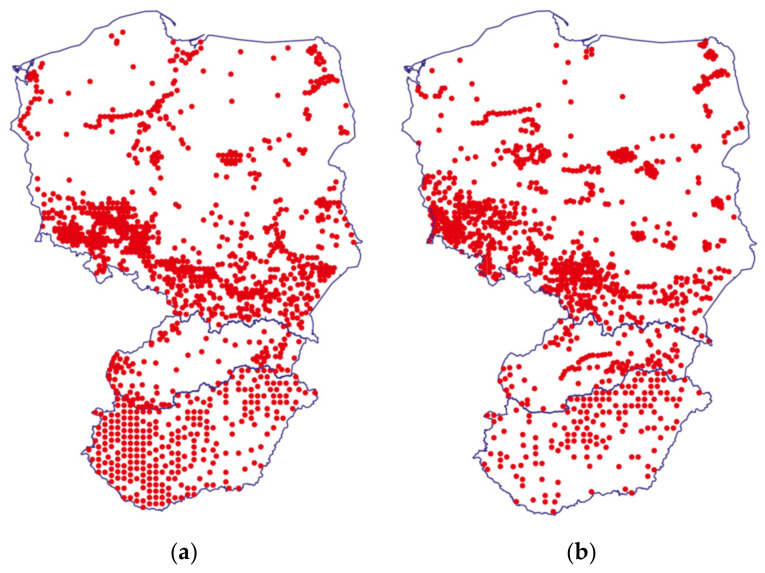
Geographical distribution (from North to South: Poland, Slovakia, and Hungary) of *Solidago gigantea* (**a**) and *Solidago canadensis* (**b**), based on [3,4,5,6,7], original illustration by Szilvia Czigle.

Representatives of the *Solidago* L. genus (Asteraceae) [1], commonly called goldenrods, are honey-bearing plants, being attractive sources of nectar for bees [2]. Among the species that belong to this genus, some are native to Europe (e.g., *S. virgaurea* L.—European goldenrod), and some of them originate from North America—northeastern United States and southern Canada (e.g., *S. canadensis* L.—Canadian goldenrod; *S. gigantea* Aiton—giant goldenrod). *S. canadensis* and *S. gigantea* were introduced to Europe and became invasive plants in several European countries. Both of these alien species are widely distributed in Hungary [3], Slovakia [4], and Poland [5,6,7] (Figure 1).

Traditionally goldenrod species have been used as melliferous and dyeing plants [2,8]. Goldenrod flowers can be the source of unifloral honey, with a light to dark amber color and strong spicy taste [2]. *Solidago* flowers offer nectar for honeybees in the late summer and early fall period, when other nectar sources are becoming rare [9]. The aerial part of *S. canadensis* is known in European traditional medicine for its use as a painkiller, antipyretic, antiemetic, sedative, and antidiarrheal drug, as well as for colds, toothaches, and burns. *S. gigantea* is used as a diuretic, for pertussis, asthma, dementia, and skin diseases [10,11]. It is also used for kidney and blood pressure problems in the ethnoveterinary medicine in North America [12]; for asthma, rheuma [13], diarrhea, bladder stones, as a carminative and antiseptic drug in India [14]; prostate diseases [15] and depression in Serbia [16]; fresh leaves for wounds in Bosnia and Herzegovina, and Montenegro in the human ethnomedicine [17]. According to the EMA monograph, only *S. virgaurea* is traditionally available for urinary complaints as an adjuvant in treatment of minor urinary complaints (to increase the amount of urine) [18].

Official herbal drugs in the European Pharmacopoeia (Ph. Eur.), 10th edition, are *Solidaginis herba* (whole or cut, dried, flowering aerial parts of *S. gigantea* or *S. canadensis*, their varieties or hybrids, and/or mixtures of these) and Solidaginis virgaureae herba (whole or fragmented, dried, flowering aerial parts of *S. virgaurea*) [19,20]. The major biologically active compounds of the three *Solidago* species above are triterpene saponins (solidagosaponins, giganteasaponins, and virgaureasaponins, thus bidesmosidics of bayogenin) and flavonoids (quercetin, kaempferol, and izorhamnetin, as well as its glycosides, such as quercitrin, isoquercitrin, hyperoside, rutin, and astragalin) [10]. The minor biologically active compounds are phenolic glycosides (leiocarposide and virgaureoside), monoterpenes (α- and β-pinene, β-myrcene, limonene, and sabinene), and diterpenes (germacrene D and cadinene) [10,11].

Honey is a natural source of bioactive compounds with antioxidant activity. Honey’s antioxidant activity provides beneficial therapeutic properties in the treatment of conditions caused by oxidative stress [21,22,23,24,25,26,27]. Antioxidant activity is a characteristic attribute of the presence of total polyphenols, flavonoids, and phenolic acids. The composition of honey and related antioxidant activity depends on various factors, such as the plant species, geographical location of the collected honey sample, and climatic conditions [24].

The aim of this work was to determine and compare the antioxidant capacity of goldenrod honeys collected in three central European countries (Hungary, Poland, and Slovakia), from three different locations within each country. The antioxidant activity was determined by using three different spectrophotometric methods: 1,1-diphenyl-2-picrylhydrazyl (DPPH), 2,2-azinobis-(3-ethylbenzothiazoline-6-sulfonate) (ABTS) also known as Trolox equivalent antioxidant capacity (TEAC), and Ferric Reducing Antioxidant Power (FRAP). We were also interested in the congruence and discriminating power of the applied spectrophotometric methods, as well as a comparison with content of phenolic compounds.

## 2. Results

Generally, at least 45% of the characteristic pollen type is required to classify a honey as unifloral, if not specified differently [28]. Since there are no generally accepted pollen frequency limit values for goldenrod honeys, we considered honey samples with at least 40% *Solidago* pollen a true goldenrod honey, if this was the most abundant pollen type [21]. Pollen analysis showed that, in this study, eight out of nine honey samples were dominated by *Solidago* pollen (more than 40%) and could, thus, be treated as unifloral goldenrod honeys. The only exception was a Slovakian honey sample (SK02), in which *Solidago* pollen was present only as minor pollen and dominated by *Robinia* pollen (Table 1).

Comparison of our samples with the color intensity of the Pfund scale showed that *Solidago* honeys from Poland had a water white color, while the color of the honey samples from Hungary and Slovakia ranged between water white and light amber (Table 2).

The combination of non-enzymatic antioxidant assays provides the most reliable results; therefore, three different total antioxidant capacity (TAC) methods [29]—DPPH, ABTS, and FRAP—were used to determine the antioxidant behaviour of the *Solidago* honey samples. For the DPPH and ABTS assays, results were expressed as SC_50_—concentration of the sample extract providing 50% inhibition of a free radical. The lower the SC_50_ value, the higher the antioxidant activity. Results were compared with ascorbic acid and Trolox solutions. For the FRAP assay, the results were expressed as the analogical amount of ascorbic acid (AA) at the initial sample concentration of 150 mg/mL, as well as compared with hyperoside (at the initial sample concentration of 20 mg/mL). The higher the AA value, the higher the antioxidant activity. The SC_50_ and AA values are summarized in Table 3.

The antioxidant activities are SC_50_ = 176.78–772.87 g/mL (DPPH), SC_50_ = 107.68–1090.31 mg/mL (ABTS), and AA = 7.03–31.61 µmol/L (FRAP) (Table 3).

The highest radical-scavenging activity was identified for Hungarian samples (*p* < 0.001), with all three antioxidant capacity assays. The DPPH and ABTS assays discriminated Polish-origin honeys with the lowest antioxidant activity (*p* < 0.001), while, according to the FRAP assay, the Slovak and Polish samples did not significantly differ from each other (*p* > 0.05).

The radical-scavenging activity was different not only in honey samples from various countries but also in various honey samples within one country. Among Hungarian-origin honeys, the highest radical-scavenging activity was identified for honey sample HU03 (Csikóstőttős), whereas, among the Slovakian samples, SK03 (Baloň), and Polish samples, PL02 (Kraków) had the lowest values (Table 3).

The content of some biologically active compounds were quantified by spectrophotometric methods—total polyphenols with Folin–Ciocalteu’s reagent, flavonoids with AlCl_3_ reagent and phenolic acids with Arnow’s reagent.

The content of total polyphenols (expressed as gallic acid equivalent) in the honey samples varied from 1.19 ± 0.12% to 6.16 ± 0.54%to a large extent (Table 4), being different in various countries (*p* < 0.001); but within Hungary and Slovakia we did not find any differences between the honey samples originating from the same country. Nevertheless, the content of polyphenols was significantly higher for the Kolbuszowa (PL03) honey sample, compared to the other two Polish-origin samples.

The content of flavonoids (expressed as hyperoside equivalent) varied from 0.53 ± 0.04% to 2.21 ± 0.15%. It was different in various countries (*p* < 0.001): the highest flavonoids content was found in Hungarian-origin honeys, while the lowest content was found in Polish-origin honey samples. The highest flavonoid content was measured for a Hungarian-origin honey, Csikóstőttős (HU03); however, among the Slovak samples, Mužla (SK02) honey had the highest value. We found no significant difference between the Polish honey samples (Table 4).

Similarly to the content of polyphenols and flavonoids, the content of the phenolic acids (expressed as caffeic acid equivalent) was significantly different among the countries (*p* < 0.001). The highest phenolic acids content was detected in Hungarian-origin honeys, while the lowest content was found in Polish-origin honey samples. The highest phenolic acids content was measured for a Hungarian-origin honey, Csikóstőttős (HU03), and the lowest one was measured in the case of a Polish-origin honey, Kraków (PL02) (Table 4).

A correlation was found between antioxidant activity and content of total polyphenols. The best correlation was found between polyphenol content and antioxidant capacity, determined with FRAP method (Table 5).

## 3. Discussion

The analysis involved a total of nine goldenrod honey samples obtained from beekeepers from nine locations across three states located in central Europe: Hungary, Slovakia, and Poland. In these countries the *Solidago* genus is present in large numbers in various locations, mostly near rivers and railway tracks and in wet meadows and marshes. The abundance of goldenrods ensures ample nectar to produce honey. Pollen analysis showed that in this study eight out of nine honey samples were dominated by *Solidago* pollen. The *Solidago* grains, which are typically 15–20 μm in diameter, isopolar, with circular outline, spheroidal shape, tricolporate aperture system, echinate and perforated exine. The study of Bonciu [30] described that the pollen grains diameter and pollen viability values were approximately similar to native and foreign genotypes of plants.

Antioxidant capacity cannot be determined by one official method and none of the available methods are perfect, because each method is suitable for the determination of a different group of antioxidants [24,25,26,27]. For this reason, we have chosen three standard in vitro methods to determine the antioxidant capacity of our honey samples —DPPH, ABTS, and FRAP. The antioxidant activity of goldenrod honeys from different countries and even from different regions of the same country was found to be significantly different. The antioxidant activity of Hungarian-, Slovak- and Polish-origin goldenrod honeys in decreasing order: HU3 (Csikóstőttős) > HU2 (Nyárád) > HU1 (Osli); SK02 (Mužla) > SK01 (Kechnec) > SK03 (Baloň); and PL03 (Kolbuszowa) > PL01 (Mikołów) > PL02 (Kraków), respectively.

The study of Dżugan et al. [24] determined the antioxidant activity of 20% (*m*/*v*) Polish goldenrod honey solutions. The scavenger activity of 11 samples, determined with DPPH assay and expressed as % of inhibition, ranged from 22.49% to 82.47% [24]. Similar results were obtained in Jasicka-Misiak et al.’s [31] study, where 20% (*m*/*v*) solutions of Polish goldenrod honeys showed 31.1% to 40.0% (DPPH) and 46.7% to 56.9% (ABTS) antioxidant potential. Compared with the previous studies [24,29], our Polish samples exhibited lower antioxidant activity (DPPH: 15.23–20.71% and ABTS: 9.22–18.90%), while the Hungarian honey samples exhibited comparable results or even higher values.

The highest antioxidant capacity was determined in Hungarian-origin honey from Csikóstőttős (HU03), which was also the sample with the darkest color (light amber) and highest content of flavonoids and phenolic acids. Higher antioxidant capacity measured for Hungarian honey samples can be caused by the geographical and/or botanical origin of honey. Goldenrod honeys from more southern locations (Hungary) exhibited higher antioxidant activity than the Polish and Slovak ones with the northernmost origin. Microclimatic differences may contribute to increased total polyphenolic content, which, in turn, influences antioxidant potential [32].

The other important factor behind the differences in the radical scavenging potential of honey samples from different regions may be the different plant origin of honeys. In Poland, goldenrod honey is produced mainly from the nectar of *S. virgaurea* [30], whereas, in Hungary, *S. gigantea* is the major source of this unifloral honey. A Polish study confirmed that the nectar producing potential of different *Solidago* species can be significantly different, affecting both nectar volumes and sugar concentrations [33]. Different botanical origin can influence not only the quality of nectar, but also the characteristics of the honey derived from a particular *Solidago* species. Jasicka-Misiak et al. [31] characterized the color of Polish goldenrod (*S. virgaurea*) honey as extra light amber, being of lighter color than the (dark) amber color of the other goldenrod honeys derived of *S. gigantea.* Similarly, in the present study, the color of Polish honey samples was the lightest, compared to Slovakian and Hungarian goldenrod honeys. In accordance with several other studies examining the relationship between honey color and antioxidant capacity [31,32,34,35,36,37,38], we found that color intensity correlated with total polyphenolic, flavonoid, and phenolic acid content, as well as antioxidant activities.

## 4. Materials and Methods

### 4.1. Honey Samples

**Figure 2:**
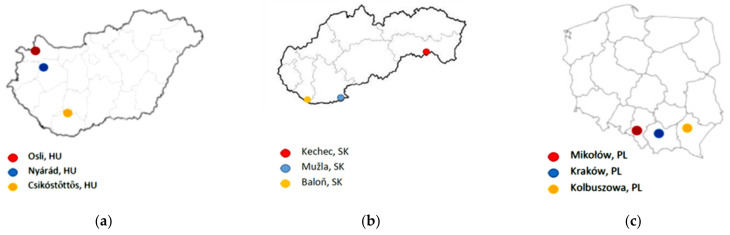
Place of origin of Hungarian- (**a**), Slovak- (**b**), and Polish-origin (**c**) goldenrod honeys.

**Figure 3:**
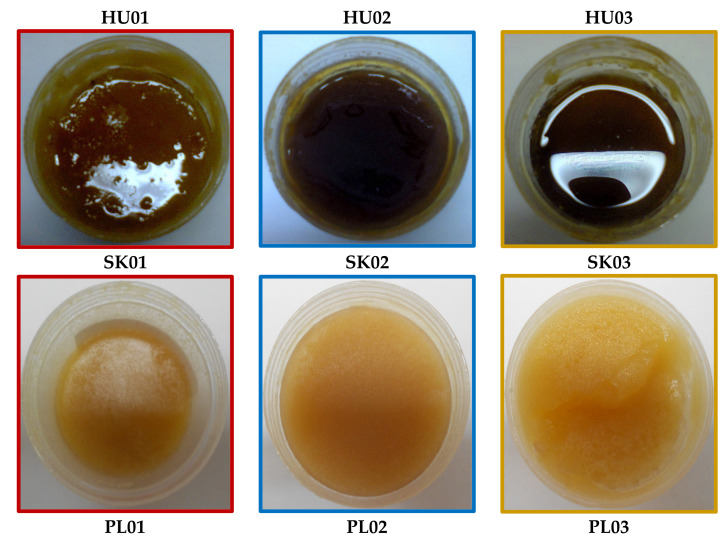

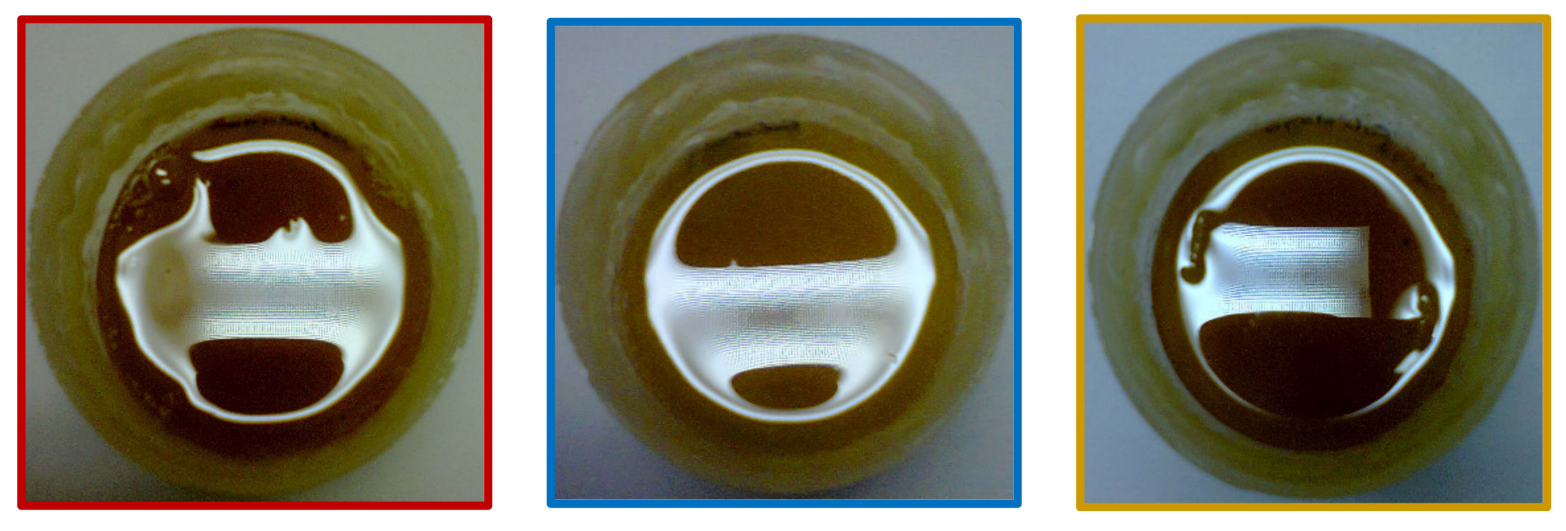
Hungarian- (HU01–HU03), Slovak- (SK01–SK 03), and Polish-origin (PL01–PL03) goldenrod honeys.

Goldenrod honeys were collected from three locations each in Hungary, Slovakia, and Poland. Hungarian-origin goldenrod honeys were collected from Osli (HU01), Nyárád (HU02), and Csikóstőttős (HU03); Slovak-origin goldenrod honeys were collected from Kechnec (SK01), Mužla (SK02), and Baloň (SK03); Polish-origin goldenrod honeys were collected Mikołów (PL01), Kraków (PL02), and Kolbuszowa (PL03) (Figure 2 and Figure 3). The samples were harvested in August–September 2018 and stored at room temperature (21 °C) in the dark until analysis.

### 4.2. Melissopalynological Analysis

The honey samples were purchased in 2018, directly from beekeepers, who identified the samples as goldenrod honeys. The botanical origin of each honey sample was checked with microscopic pollen analysis. Honey samples, when fluid, were stirred thoroughly. In case they contained large crystals, they were heated on a 40 °C water bath, until fluid, then stirred. A total of 10 g of honey was measured into 50 mL centrifuge tubes; 20 mL distilled water was added, then vortexed with Combi-spin FVL-2400N (Biocenter Ltd., Szeged, Hungary). The solution was centrifuged with 3000 rpm for 10 min with a Neofuge 15R centrifuge (Lab-Ex Ltd., Budapest, Hungary). The supernatant was decanted, then 10 mL distilled water was added to the sediment; this mixture was centrifuged again with 3000 rpm for 10 min and decanted. Any remaining fluid was removed by setting the centrifuge tubes on filter paper. A frame, of the size of the cover glass, was drawn on each microscope slide with a paint marker (Edding 750, D. Ledermann & Co. GmbH, Bautzen, Germany); then, the microscope slides were placed on a heating plate (OTS 40, Tiba Ltd., Győr, Hungary) set to 40 °C. A total of 0.25 mL distilled water was added to the sediment in the centrifuge tube, then vortexed. A volume of 20 μL of the pollen suspension was pipetted on the microscope slide within the frame. Water was allowed to evaporate from the slide on the heating plate. The pollen preparation was mounted in fuchsine glycerol jelly (fuchsine added to Kaiser’s glycerol jelly). Pollen preparations were studied with a Nikon Eclipse E200 microscope equipped with a Michrome 20MP CMOS digital camera (Auro-Science Consulting Ltd., Budapest, Hungary), and microphotos were taken with the software Capture 1.2 at 400× magnification. At least 500 pollen grains per honey sample were counted, and the source plants were identified at the species, or at least family, level. The relative frequency for each type of pollen was calculated as the percentage of the total number of pollen grains [39].

### 4.3. Color Intensity

Color intensity was determined according to Ferreira et al. [40]. *Solidago* honey samples were diluted to 50% (*m*/*v*) with distilled water, mixed, and centrifuged at 3200 rpm/5 min (centrifuge Hettich Universal 320 R, Hettich GmbH&Co.KG, Darmstadt, Germany). The absorbance was measured at 635 nm using a Genesys^TM^10 spectrophotometer (Thermo Electron Corporation, Cambridge, UK), and color intensity [41] was determined using the Pfund scale, using the following equation:

Pfund scale = −38.70 + 371.39 × A

A = absorbance.

### 4.4. DPPH Method

The DPPH assay was conducted according to the method reported by Vundać et al. [42]. A total of 1.8 mL of DPPH methanol solution was added to 0.2 mL of various concentrations of honey extracts. The solution was then thoroughly shaken and left to react in the dark at room temperature. The absorbance of the solution was measured after 30 min. Methanol (1.8 mL) and honey extracts (0.2 mL) were used as blank; DPPH solution (1.8 mL) and methanol (0.2 mL) were used as negative control. The positive control was 1.8 mL of DPPH solution and 0.2 mL of ascorbic acid/Trolox solution. Antioxidant activity (%) was calculated using the samples’ vs. negative control’s absorption values at 517 nm (Genesys^TM^10 spectrophotometer, Thermo Electron Corporation, Cambridge, UK), and results were expressed as SC_50_ (concentration of sample extract providing 50% inhibition of the DPPH radical). The assay was carried out in triplicate [29].

### 4.5. ABTS Method

The ABTS assay was conducted according to the method reported by Re et al. [43]. A total of 2 mL of ABTS radical solution was added to 0.1 mL of various concentrations of honey extracts. The solution was then thoroughly shaken and left to react in the dark at room temperature. The absorbance of the solution was measured after 5 min. Ethanol (2 mL) and honey extracts (0.1 mL) were used as blank; ABTS solution (2 mL) and methanol (0.1 mL) were used as negative control. The positive control was 2 mL of ABTS solution and 0.1 mL of ascorbic acid/Trolox solution. Antioxidant activity (%) was calculated using the samples’ vs. negative control’s absorption values at 734 nm (Genesys^TM^10 spectrophotometer, Thermo Electron Corporation, Cambridge, UK), and results were expressed as SC_50_ (concentration of sample extract providing 50% inhibition of the ABTS radical). The assay was carried out in triplicate [29].

### 4.6. FRAP Method

The FRAP assay was conducted according to the method reported by Benzie and Strain [27]. A total of 3 mL of the FRAP reagent was added to 0.1 mL of various concentrations of honey extracts. The solution was then thoroughly shaken and left to react in the dark at room temperature. The absorbance of the solution was measured after 5 min. The FRAP reagent was used as blank. Hyperoside was used as positive control. Results were expressed as an analogical amount of ascorbic acid (μg/mL) and calculated using the samples’ absorption values at 593 nm (Genesys^TM^10 spectrophotometer, Thermo Electron Corporation, Cambridge, UK). The assay was carried out in triplicate [29].

### 4.7. Quantification of Total Polyphenolic Compounds Expressed as Gallic Acid

The quantification of total polyphenols was performed following the analytical procedure described by Singleton et al. [44]. This method is a modified spectrophotometric Folin–Ciocalteu’s method. Briefly, 5 g of honey was mixed with distilled water up to 10.0 mL. Then, 1.0 mL (50% *m*/*v*) of honey extract was mixed with 1.0 mL of Folin–Ciocalteu’s reagent. In 3 min, 1.0 mL of 10.0% (*m*/*v*) Na_2_CO_3_ solution was added to the mixture and adjusted to 10.0 mL with distilled water. The reaction was kept in the dark for 90 min, after which the absorbance was read at 725 nm using a Genesys^TM^10 spectrophotometer (Thermo Electron Corporation, Cambridge, UK). The results are reported as the mean ± standard deviation and expressed as the % of gallic acid from a calibration curve (k = 23.4). All samples were analyzed in triplicate.

### 4.8. Quantification of Flavonoid Expressed as Hyperoside

The quantification of flavonoids was performed following the modified spectrophotometric analytical procedure [45]. For the quantification of flavonoids, honey samples were diluted to 50% (*m*/*v*). Firstly, 1.0 mL of honey extract was mixed with a solution 1.0 mL of hexamethylenetetramine (5 g/L), 20.0 mL of acetone, and 2.0 mL of hydrochloric acid, and the mixture was boiled for 30 min. After phase–phase separation with ethyl acetate, the combine ethyl acetate extract added 1.0 mL of 10% (*m*/*v*) AlCl_3_ reagent and diluted to 25.0 mL with a 5% (*v*/*v*) solution of glacial acetic acid in methanol. After 30 min, the absorbance was measured at 425 nm using a Genesys^TM^10 spectrophotometer (Thermo Electron Corporation, Cambridge, UK). The results are reported as the mean ± standard deviation and expressed as the % of hyperoside from a calibration curve (k = 34.5). All samples were analyzed in triplicate.

### 4.9. Quantification of Phenolic Acid Expressed as Caffeic Acid

For the quantification of phenolic acids expressed as caffeic acid, according to Arnow’s method [46], we used diluted honey samples (50% *v*/*w*). Firstly, 1.0 mL of honey ethanolic extract was mixed with 2.0 mL of 0.5 M hydrochloric acid, 2.0 mL of Arnow’s reagent (containing NaNO_2_), and 2 mL NaOH (8.5 g/100 mL). The volume was increased to 10 mL with distilled water. The mixture was shaken, and the absorbance was read at 505 nm using a Genesys^TM^10 spectrophotometer (Thermo Electron Corporation, Cambridge, UK). The results are reported as the mean ± standard deviation and expressed as the % of caffeic acid from a calibration curve (k = 44.5). All samples were analyzed in triplicate.

### 4.10. Statistical Analysis

All measurements were completed on three biological replicates of nine *Solidago* honey samples. The data were compared with one-way ANOVA with Tukey’s pairwise comparisons. If the normality assumption was violated, we applied Kruskal–Wallis test with Mann-Whitney pairwise comparisons. Differences were considered statistically significant at *p* ≤ 0.05. Statistical analyses were carried out using PAST software package version 2.17b, after normality checking with the Shapiro–Wilk test.

Pearson’s correlation coefficient was measured to find the association between color intensity, total polyphenolic content, flavonoid content, phenolic acid content, and antioxidant activities using Statistica 14.0.

## 5. Conclusions

This study examined the antioxidant capacity and bioactive compounds of nine goldenrod honeys from three countries, out of which, eight samples were identified as true unifloral honeys. The antioxidant capacity (measured with DPPH, ABTS, and FRAP methods) of *Solidago* honeys correlated with color intensity and the content of phenolic compounds (total polyphenols, flavonoids, and phenolic acids). Our results suggest that all of the investigated honey traits are influenced by both the botanical and geographical origin of honey. Our findings revealed that even unifloral honeys originating from the same plant genus (*Solidago*), but not necessarily from the same species, can differ regarding their bioactivity, which showed an increasing tendency from the northern towards the southern regions within central Europe.

## Figures and Tables

**Table 1:** Pollen analysis of Hungarian-, Slovak-, and Polish-origin goldenrod honey.

Sample Code	Geographical Origin	Country	Pollen Type—Relative Frequency (%) ^a^					
			*Solidago*	*Robinia*	*Brassica*	*Taraxacum*	*Helianthus*	Other
HU01	Osli	Hungary	47.61	2.38	-	-	40.47	9.52
HU02	Nyárád	Hungary	40.50	5.30	3.03	-	3.03	48.10
HU03	Csikóstőttős	Hungary	40.11	4.93	-	-	2.69	52.09
SK01	Kechnec	Slovakia	70.58	-	-	-	9.80	19.60
SK02	Mužla	Slovakia	1.53	31.28	-	-	1.84	57.66
SK03	Baloň	Slovakia	42.85	17.85	-	-	14.28	25.00
PL01	Mikołów	Poland	57.01	1.86	3.33	4.93	-	32.84
PL02	Kraków	Poland	49.98	4.59	4.26	0.32	-	40.78
PL03	Kolbuszowa	Poland	84.02	0.27	0.82	-	-	14.87

^a^ Evaluation of pollen samples: predominant pollen: >45% of the pollen grains counted; secondary pollen: 16–45%; important minor pollen 3–15%; minor pollen <3%.

**Table 2:** Color intensity of Hungarian-, Slovak-, and Polish-origin goldenrod honey.

Sample Code	Geographical Origin	Country	Weight [g]	A	Pfund Scale ^a^ (Color Intensity)	Color Name
HU01	Osli	Hungary	2.5023	0.254	55.63	light amber
HU02	Nyárád	Hungary	2.5008	0.177	27.04	white
HU03	Csikóstőttős	Hungary	2.5049	0.325	82.00	light amber
SK01	Kechnec	Slovakia	2.5025	0.197	34.46	extra light amber
SK02	Mužla	Slovakia	2.5052	0.208	38.55	extra light amber
SK03	Baloň	Slovakia	2.4996	0.120	5.87	water white
PL01	Mikołów	Poland	2.5011	0.124	7.35	water white
PL02	Kraków	Poland	2.5034	0.108	1.41	water white
PL03	Kolbuszowa	Poland	2.5031	0.105	0.30	water white

^a^ Pfund Scale (mm): water white <9; extra white 9–17; white 18–34; extra light amber 35–50; light amber 51–85; amber 86–114; dark amber >114.

**Table 3:** Antioxidant activity of Hungarian-, Slovak-, and Polish-origin goldenrod honey.

Sample Code	Geographical Origin	Country	DPPH ^a^ SC_50_ (mg/mL)	ABTS ^b^ SC_50_ (mg/mL)	FRAP ^c,d^ (µmol/L)
HU01	Osli	Hungary	392.39 ± 11.24 *	381.66 ± 12,22 **	13.22 ± 0.23 *
HU02	Nyárád	Hungary	302.18 ± 10.22 *	308.38 ± 10,10 *	13.81 ± 0.20 *
HU03	Csikóstőttős	Hungary	176.78 ± 6.88 ***	107.68 ± 6.04 ***	31.61 ± 1.01 **
SK01	Kechnec	Slovakia	328.84 ± 12.04 *	329.97 ± 12.45 *	12.46 ± 0.56 *
SK02	Mužla	Slovakia	317.46 ± 11.22 *	300.05 ± 10.28 *	13.05 ± 0.32 *
SK03	Baloň	Slovakia	548.77 ± 14.66 **	482.25 ± 12.22 **	7.03 ± 0.23 **
PL01	Mikołów	Poland	510.78 ± 13.65 *	566.88 ± 13.88 *	7.71 ± 0.28 *^,^**
PL02	Kraków	Poland	772.87 ± 25.88 **	1 090.31 ± 45.22 **	7.12 ± 0.57 **
PL03	Kolbuszowa	Poland	463.51 ± 12.22 ***	527.68 ± 14.11 ***	8.73 ± 0.24 *^,^***
ascorbic acid			0.02 ± 0.00	0.02 ± 0.00	–
Trolox			0.02 ± 0.00	0.28 ± 0.00	–
hyperoside			–	18.44 ± 1.42	5.44 ± 0.42 ^e^

^a^ DPPH—antiradical power; ^b^ ABTS—Trolox equivalent antioxidant capacity; ^c^ FRAP—ferric reducing antioxidant power. Data are means ± standard deviations of three independent determinations (*n* = 3); data in the same column with different superscripted symbols (*, **, ***) mean significant differences among honeys within one country: * *p* < 0.05, ** *p* < 0.01, *** *p* < 0.001; ^d^ Ascorbic acid (AA) value at the initial sample concentration of 150 mg/mL; ^e^ AA value at the initial sample concentration of 20 mg/mL [29].

**Table 4:** Content of total polyphenols, flavonoids, and phenolic acids of Hungarian-, Slovak-, and Polish-origin goldenrod honey.

Sample Code	Geographical Origin	Country	Total Polyphenols as Gallic Acid (%)	Flavonoids as Hyperoside (%)	Phenolic Acids as Caffeic Acid (%)
HU01	Osli	Hungary	1.19 ± 0.12 *	1.07 ± 0.10 *	0.55 ± 0.04 *
HU02	Nyárád	Hungary	1.54 ± 0.12 *	0.87 ± 0.06 *	0.48 ± 0.04 *
HU03	Csikóstőttős	Hungary	1.51 ± 0.13 *	2.21 ± 0.15 **	1.76 ± 0.10 **
SK01	Kechnec	Slovakia	2.12 ± 0.18 *	0.86 ± 0.08 **	0.46 ± 0.04 *
SK02	Mužla	Slovakia	2.11± 0.18 *	0.89 ± 0.07 *	0.45 ± 0.04 *
SK03	Baloň	Slovakia	1.61 ± 0.13 *	0.59 ± 0.06 **	0.39 ± 0.04 *
PL01	Mikołów	Poland	2.37 ± 0.19 *	0.56 ± 0.04 *	0.36 ± 0.03 *
PL02	Kraków	Poland	2.61 ± 0.18 *	0.53 ± 0.04 *	0.23 ± 0.01 **
PL03	Kolbuszowa	Poland	6.16 ± 0.54 **	0.62 ± 0.05 *	0.32 ± 0.03 *

Data are means ± standard deviations of three independent determinations (*n* = 3). Data in the same column with different superscripted symbols (*, **) mean significant differences among honeys within one country: * *p* < 0.05, ** *p* < 0.01.

**Table 5:** Correlation between color intensity, total polyphenols, flavonoids, and phenolic acids content and antioxidant activities.

Antioxidant Methods	Pearson’s Correlation Index (r) with			
	Color Intensity	Total Polyphenols as Gallic Acid (%)	Flavonoids as Hyperoside (%)	Phenolic Acids as Caffeic Acid (%)
DPPH	0.446591	−0.719830	0.270690	0.203474
ABTS	0.534654	−0.661663	0.323427	0.229375
FRAP	−0.471335	0.988781	−0.322422	−0.299204

## Data Availability

The data presented in this study are available on request from the corresponding author.
